# Dietary Galacto-Oligosaccharides Enhance Growth Performance and Modulate Gut Microbiota in Weaned Piglets: A Sustainable Alternative to Antibiotics

**DOI:** 10.3390/ani15111508

**Published:** 2025-05-22

**Authors:** Yongchao Wang, Zhong Li, Guowu Chen, Yiyuan Xing, Jingjing Wang, Yujie Zhao, Meng Kang, Ke Huang, Enkai Li, Xiaokang Ma

**Affiliations:** 1 College of Animal Science and Technology, Hunan Agricultural University, Changsha 410128, China; 13484693881@163.com (Y.W.); 13054060618@163.com (Z.L.); 18074402162@163.com (G.C.); 15816154370@163.com (Y.X.); zijinjin0803@163.com (J.W.); 1609529035@163.com (Y.Z.); huangke5526@edu.cn (K.H.); 2 Yuelushan Laboratory, Changsha 410128, China; 3 Department of Animal Sciences, Purdue University, West Lafayette, IN 47907, USA; lienkai1994@gmail.com

**Keywords:** galacto-oligosaccharides, growth performance, gut microbiota, intestinal morphology, antibiotics, weaned piglets

## Abstract

Galacto-oligosaccharides (GOS) are recognized prebiotics with extensive applications in animal nutrition. This study aimed to elucidate the effects of chlortetracycline (CTC) supplementation alongside GOS on growth performance, intestinal morphology, and microbiota composition in weaned piglets. A total of 72 weaned piglets [(Duroc × (Landrace × Yorkshire)] with an initial body weight of 7.64 ± 0.15 kg were randomly assigned to three dietary treatments: a corn–soybean meal-based diet (CON), a corn–soybean meal-based diet supplemented with chlortetracycline (CTC) at 75 mg/kg, and a diet with 1500 mg/kg GOS. The experiment spanned 14 days, divided into early (1–7 days) and late (7–14 days) stages. The results indicated that the GOS group exhibited a significant increase in body weight (BW) by day 14 and improved average daily gain (ADG) from day 1 to 14, alongside a decreased feed-to-gain ratio and diarrhea incidence (*p* < 0.05). Notably, GOS supplementation enhanced the villus height of the jejunum and increased the ratio of villus height to crypt depth compared to the CON group, although no significant differences were observed between GOS and AntB groups. Additionally, the number of goblet cells in the colonic crypts increased significantly with GOS supplementation. High-throughput 16S rRNA gene sequencing revealed an increased relative abundance of Firmicutes in the GOS group, coupled with a decrease in Bacteroidetes and Spirochaetota. Beneficial bacteria such as Lactobacillus and Faecalibacterium were significantly enriched, while potentially pathogenic bacteria, including Clostridium_sensu_stricto_1 and Treponema, were reduced. In summary, dietary GOS supplementation can enhance beneficial microbiota while diminishing harmful species, thereby improving intestinal morphology and growth performance in weaned piglets. Consequently, GOS emerges as a promising alternative to in-feed antibiotics in contemporary animal husbandry.

## Simple Summary

The extensive use of antibiotics in weaned piglets has historically aimed to enhance growth and prevent infections. However, this practice poses critical risks rooted in fundamental principles of public health and sustainability. The overuse of antibiotics has led to the emergence of antimicrobial resistance, posing severe risks to human health through resistant pathogens and residues in animal products. Additionally, antibiotics can cause harmful changes in the intestinal flora. This investigation aimed to elucidate the effects of dietary galacto-oligosaccharides (GOS) as a prospective alternative to antibiotics concerning the growth performance, intestinal morphology, and microbiota of weaned piglets. The findings demonstrated that the incorporation of GOS into the diet of weaned piglets significantly enhanced their growth metrics and concurrently diminished the incidence of diarrhea. Furthermore, GOS was found to promote intestinal health by augmenting the prevalence of beneficial bacterial populations while concurrently reducing pathogenic strains, thereby fostering a balanced intestinal flora composition. This study underscores the potential of GOS as a viable dietary intervention to bolster the health and growth of weaned piglets in agricultural practices.

## 1. Introduction

Weaning piglets in the contemporary swine industry encounter substantial post-weaning stresses that significantly impact their health and productivity. These stresses arise from alterations in diet, social dynamics, and environmental conditions. Such factors contribute to an increased risk of disease and mortality, diminished growth rates, and disruptions to the intestinal microbiota. In Europe, it has been reported that 17% of piglets perish each year during the weaning phase due to infections from opportunistic pathogens [1]. As the leading global producer and consumer of pork, China faces a pressing challenge, with an alarming estimate of approximately 24 million post-weaning piglets succumbing annually to diarrheal diseases [2]. This considerable mortality rate is predominantly attributed to inadequate management practices during the critical transition period of weaning, which poses severe economic repercussions for pork production systems.

Historically, antibiotics have been employed extensively in weanling piglets to enhance growth and mitigate infections [3,4,5]. However, the overuse of antibiotics has culminated in the emergence of resistance and the presence of antibiotic residues within livestock and poultry, which poses significant threats to human health [6]. Additionally, the broad-spectrum antibacterial properties of antibiotics can lead to detrimental alterations in the intestinal flora [7]. The rise of antibiotic resistance, coupled with the occurrence of antimicrobial residues, presents formidable challenges in the management of pathogenic bacterial infections [8]. For instance, apramycin sulfate has been widely utilized in China for the prophylaxis of piglet diarrhea, yet its application may induce cross-resistance between apramycin and gentamicin in Escherichia coli and Salmonella enteritidis [9]. Consequently, the European Commission enacted a regulatory prohibition in January 2006, discontinuing the use of antimicrobial agents as zootechnical feed additives due to the associated risks of propagating antibiotic resistance [10]. In response, several nations have implemented or are in the process of enacting bans on the incorporation of antibiotics into pig diets as a standard growth promotion strategy. Notably, China has prohibited colistin sulfate as a feed additive since April 2017, followed by Argentina’s ban in February 2019 [11]. The transition to a comprehensive ban on antibiotics necessitates urgent action to ensure the safety of pork products, thereby highlighting the imperative for the establishment of antibiotic-free farming practices and the development of innovative alternatives to conventional antibiotic feed additives that are environmentally sustainable and efficacious.

Various alternatives to antibiotics have been identified that contribute to maintaining the health of pigs and enhancing growth performance, including probiotics, prebiotics, acidifiers, and essential oils [1]. Prebiotics are defined as substrates selectively utilized by host microorganisms, conferring health benefits to the host [12]. Recent research indicates that prebiotics present a superior alternative to antibiotics [13]. Currently, commercially available prebiotics encompass xylo-oligosaccharides (XOS), fructooligosaccharides [14], inulin [15], and galacto-oligosaccharides (GOS) [16]. XOS are sugar oligomers consisting of 2 to 6 xylose units connected via β-(1→4)-glycosidic linkages [17]. Fructo-oligosaccharides (FOS), on the other hand, are composed of 2 to 10 fructosyl units linked by β-(2→1)- or β-(2→6)-glycosidic bonds, and may include a terminal D-glucose residue [18].

GOS, a specific type of prebiotic, consists of two to eight saccharide units, with one terminal glucose unit and the remainder consisting of galactoses and disaccharides composed of two galactose units [19,20]. Among various oligosaccharides with potential prebiotic applications, galacto-oligosaccharides (GOS) are considered functional oligosaccharides characterized by high safety, broad consumer acceptance, and effective nutritional benefits. GOS naturally occur in animal milk and have been widely recognized for their feed efficacy [21,22,23]. Notably, several studies have reported that GOS exhibit a superior adhesion-inhibitory effect compared to other prebiotics such as lactulose, inulin, raffinose, and fructo-oligosaccharides, thereby contributing to the prevention of infections by hindering the adhesion of pathogenic microorganisms [24]. In addition, GOS have been shown to promote the production of health-associated short-chain fatty acids (SCFAs), support the growth and differentiation of colonic epithelial cells, enhance energy metabolism within colonic cells, and regulate lipid and carbohydrate metabolism. Furthermore, they contribute to reducing populations of potentially pathogenic bacteria and support the maintenance of normal intestinal function [25]. Prior studies have demonstrated that GOS can enhance intestinal health and growth performance in animals, while promoting the proliferation of beneficial gut bacteria, such as Bifidobacterium and Lactobacillus [26,27,28]. Nevertheless, the application of GOS as an antibiotic substitute in weaned piglets remains under-explored. Therefore, this study aims to investigate the effects of dietary GOS as a potential replacement for antibiotics on the growth, intestinal morphology, and microbiota of weaned piglets.

## 2. Materials and Methods

### 2.1. Ethics

This study has been reviewed and approved by the Animal Care Committee of Hunan Agricultural University (Changsha, China). All animal care procedures are carried out in accordance with the Chinese Animal Welfare Guidelines. Humane animal care was practiced throughout the experiment, and every effort was made to minimize the suffering of the piglets.

### 2.2. Animals and Experimental Design

A total of 72 healthy, castrated, male weaned piglets (Duroc × Landrace × Yorkshire), with an initial average body weight of 7.64 ± 0.15 kg, were randomly allocated to three dietary treatment groups using a completely randomized block design. Each treatment included eight replicates, with three piglets per replicate, balanced by body weight. The experimental treatments were as follows: (1) a basal corn–soybean meal diet (control group, CON); (2) the basal diet supplemented with 75 mg/kg of pure chlortetracycline (antibiotic group, AntB); and (3) the basal diet supplemented with 1500 mg/kg of galacto-oligosaccharides (GOS group, GOS). The GOS used in this study was obtained from Shandong Baolingbao Biology Co., Ltd. (Dezhou, China), a certified producer of functional oligosaccharides. The product was of food-grade quality with a minimum purity of 90% (dry basis) and was composed primarily of β-1,4 and β-1,6 galactose oligomers. Prior to the initiation of the experiment, all piglets were confirmed to be free from clinical symptoms of diarrhea or other health disorders. The experimental period lasted 14 days, comprising an early phase (days 1–7) and a late phase (days 8–14). Throughout the study, animals had ad libitum access to feed and clean drinking water. Housing conditions were carefully managed, including regular implementation of pest control and immunization measures, as well as thorough cleaning and disinfection protocols. Ambient temperature was regulated via an automated thermostatic system, and natural ventilation was periodically provided by opening windows. All experimental diets (Table 1) were provided in mash form and were formulated to meet or exceed the estimated nutrient requirements for nursery pigs, as specified by the NRC (2012) guidelines [29].

### 2.3. Sample Collection

At the end of the experiment, from each treatment group (a total of 3 groups) of 8 replicates, one healthy piglet (a total of 24, n = 8/group) with an average weight close to that of the replicate was randomly selected for euthanasia. Afterward, the entire intestine was removed from each pig. Segments of the jejunum and colon flushed with saline were collected. These intestinal segments were immediately fixed in 4% paraformaldehyde solution and then embedded in paraffin for morphological examination. The luminal digesta of the colon was collected aseptically into sterile plastic containers and stored at −80 °C until processing.

### 2.4. Growth Performance and Diarrhea Ratio

Daily feed intake for each replicate group of weaned piglets was recorded, and body weight measurements were used to calculate key growth performance indicators, including average daily gain (ADG), average daily feed intake (ADFI), and the gain-to-feed ratio (G:F) (feed conversion ratio). Fecal consistency was assessed visually and scored according to established criteria [30]: 0 = normal feces (well-formed strips or pellets), 1 = soft feces (formed but moist and soft), 2 = semi-liquid feces (high moisture content without separation of solids and liquid), and 3 = liquid feces (very high water content with clear separation of solids and liquid). Piglets with fecal scores of 2 or higher were classified as having diarrhea. The diarrhea incidence rate was calculated using the following formula: Diarrhea rate (%) = 100 × (number of diarrheic piglets × number of diarrhea days)/(total number of piglets × number of experimental days).

### 2.5. Morphological Examination

PAS staining was performed according to standard protocols [31]. Paraformaldehyde-fixed jejunum and colon segments taken were then dehydrated with ethanol, embedded in paraffin, and sectioned (5 µm). After dewaxing and immediately washing with distilled water for 1 min, the specimens were immersed in 0.5% periodate solution (Sigma Co., Shanghai, China) for 5 min at room temperature in the dark. Afterward, sections were immediately washed (30 s × 2) and soaked in Schiff’s solution at 37 °C. After 60 min, sections were washed twice with a sulfuric acid solution then quickly rinsed with distilled water. The subsequent steps followed the routine protocols of the laboratory. The sections were examined using light microscopy. Image J 1.43u and Image J pro plus 6.0 were employed to calculate gut-related indicators such as villus height, crypt depth and the number of goblet cells (GCs).

### 2.6. Intestinal Microbiome Structure

DNA was extracted from colonic content samples using the Stool DNA Isolation Kit (Tiangen Biotechnology Co., Ltd., Beijing, China). The V3-V4 hypervariable region of the bacterial 16S rRNA gene sequence was amplified by PCR using Illumina Miseq (PE300) with primer number 338F-806R. The 30 µL PCR reaction system included the following: 1 µL of each primer, 1 µL of DNA template, 15 µL of Phusion^®^ PCR Master Mix (New England Biolabs Co., Ltd., Beijing, China), and 12 µL of sterile water. After the PCR reaction, gel electrophoresis and GeneJET Gel Extraction Kit (Thermo Fisher Scientific, Wilmington, DE, USA) were used to detect, extract, and purify the PCR products. Sequence libraries were constructed using the NEB Next R UltraTM DNA Library Construction Kit and sequenced using the Illumina platform (New England Biolabs Co., Ltd., Beijing, China). The Illumina HiSeq platform used HiSeq2500 PE250 (Illumina, San Diego, CA, USA) for paired-end sequencing to generate 250 bp paired-end reads. Majorbio Bio-Pharm Technology Co., Ltd. (Shanghai, China) carried out the sequencing.

### 2.7. Bioinformatics Analysis

The QIIME (V1.7.0) official method was employed for classification annotation. After mass filtering, it was denoised using DaDa2 (1.26) (Split amplicon denoising algorithm 2) and the resulting high-quality amplicon sequence variants (ASVs) are used for downstream analysis. Each ASV represented a unique biological sequence present in the original sample, and taxonomic annotation was performed using the silva-138-99-nb-classifier classifier combined with a traditional Bayesian algorithm. Normalized microbiome data were used for diversity and species composition analysis, α-diversity was calculated using R software’s Vegen package (V3.2.0), and β-diversity based on the Bray-curtis distance matrix was calculated. Meanwhile, analysis of similarities (ANOSIM) was adopted to evaluate the significant difference between samples. The visualization of microbiome data was performed using R software (V3.2.0).

### 2.8. Statistical Analysis

Statistical software (SAS 9.2) was used to conduct one-way analysis of variance (ANOVA) for data analysis. *p* ≤ 0.05 was considered as significant difference, *p* ≤ 0.01 was considered as extremely significant difference, and 0.05 ≤ *p* < 0.10 was considered as trend.

## 3. Results

### 3.1. Growth Performance and Diarrhea Rate

As evidenced in Table 2, the final weight of piglets within the GOS group was significantly greater than that of the CON group (*p* < 0.01), with no notable difference observed when compared to the AntB group. In contrast to the CON group, both the GOS and AntB groups exhibited substantial enhancements in average daily gain (ADG) and a reduction in the gain-to-feed ratio (G:F) among weaned piglets (*p* < 0.05). Additionally, the incidence of diarrhea was markedly reduced (*p* < 0.05). Analyzing the periods of days 1 to 7 and days 7 to 14, the ADG in both the GOS and AntB groups showed a significant increase compared to the CON group (*p* < 0.01), while no significant variations in average daily feed intake (ADFI) were detected across all groups. Noteworthy is the significant improvement in the G:F ratio during days 7 to 14 and throughout the overall experimental period (days 1 to 14) for both the GOS and AntB groups, relative to the CON group (*p* < 0.01). However, no significant differences in ADFI or G:F ratios were found between the GOS and AntB groups at any point during the study.

### 3.2. Effects of Dietary Treatments on Colonic and Jejunal Morphology

**Figure 1:**
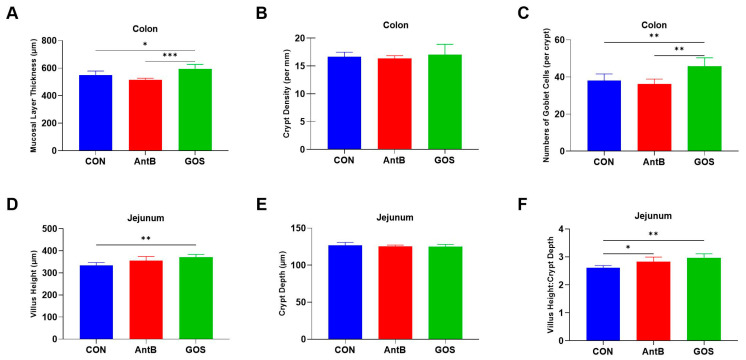
Effect of dietary treatments on histological morphology in the colon and jejunum of weaned piglets. (**A**) Mucosal layer thickness, (**B**) crypt density, (**C**) number of goblet cells, (**D**) villus height, (**E**) crypt depth, (**F**) villus height/crypt depth. CON, corn–soybean meal-based diet; AntB, corn–soybean meal-type diet + 75 mg/kg pure chlortetracycline; GOS, CON + 1500 mg/kg GOS. * *p* ≤ 0.05; ** 0.001 < *p* ≤ 0.01; *** *p* ≤ 0.001.

The effects of dietary treatments on intestinal characteristics are illustrated in Figure 1. Notably, the villus height of the jejunum exhibited a significant increase in the GOS group when compared to the CON group (*p* < 0.05), while no significant difference was observed relative to the AntB group. Furthermore, the dietary treatments did not yield significant alterations in the crypt depth of the jejunum. In addition, both GOS and AntB diets did not demonstrate significant changes in the crypt density of the colon. The ratios of villus height to crypt depth in the jejunum were significantly elevated in the GOS and AntB groups compared to the CON group (*p* < 0.05). Moreover, GOS supplementation significantly increased the number of goblet cells in the crypt of the colon and enhanced mucosal layer thickness compared to both the AntB and CON groups (*p* < 0.05).

### 3.3. Effects of Dietary Treatments on the Colonic Microbiota

**Figure 2:**
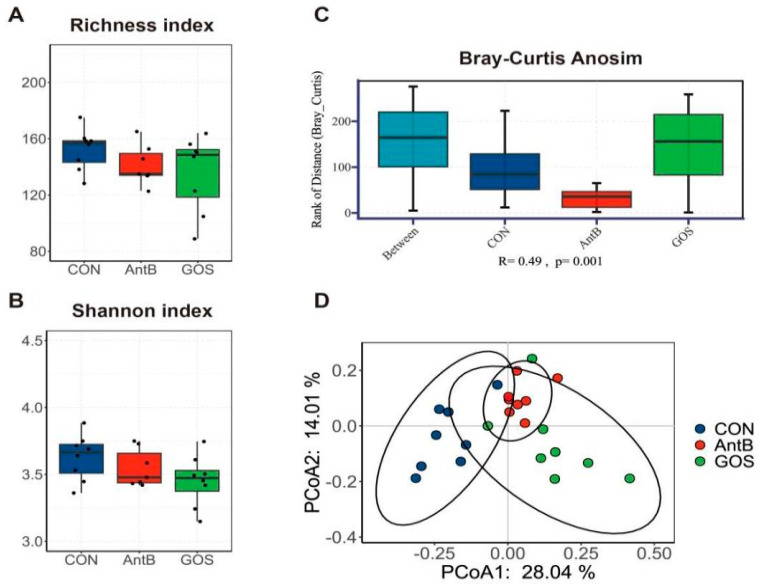
Effect of dietary treatments on colonic microbiota diversity and composition of weaned piglets. (**A**) the Richness index of colonic mucosa microbiota, (**B**) the Shannon index of colonic mucosa microbiota, (**C**) ANOSIM analysis of β-diversity, (**D**) principal coordinate analysis (PCoA) on colonic microbiota, ANOSIM analysis was used to assess significant differences. CON, corn–soybean meal-based diet; AntB, corn–soybean meal-type diet + 75 mg/kg chlortetracycline; GOS, CON + 1500 mg/kg GOS.

In this study, the 16S rRNA gene sequencing technology was employed to systematically analyze the intestinal flora across three distinct sample groups: CON, AntB, and GOS. The α-diversity analysis revealed no statistically significant differences in the Richness index and Shannon index among the groups (*p* > 0.05), suggesting comparable levels of microbial diversity (Figure 2A,B). In contrast, the β-diversity analysis, conducted using the Bray–Curtis distance matrix and ANOSIM test, identified significant structural variations in the microflora among the three sample groups (R = 0.49, *p* = 0.001, Figure 2C). This indicates that the treatments exerted significant influences on the intestinal microflora structure. A comparative analysis of the Bray–Curtis distances of colon content samples demonstrated a clear demarcation of the microbiota in the GOS group from those in the AntB and CON groups. Notably, the first axis of the Principal Coordinates Analysis (PCoA) accounted for 28.04% of the variation in bacterial diversity, while the second axis accounted for 14.01% (Figure 2D).

**Figure 3:**
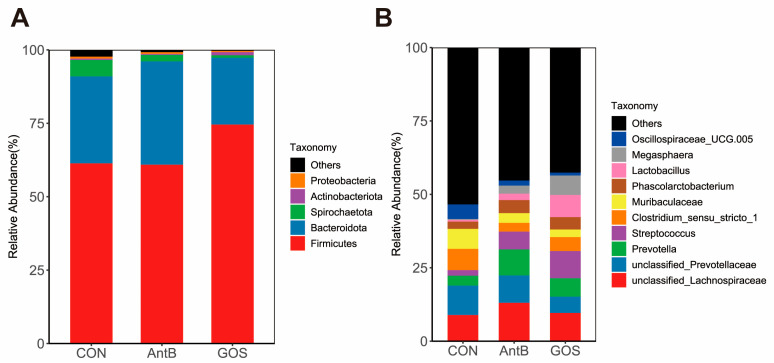
The composition of microbiota at the phylum and genus level in the colonic digesta samples of weaned piglets. (**A**) Phylum-level composition, (**B**) genus-level composition. CON, corn–soybean meal-based diet; AntB, corn–soybean meal-type diet + 75 mg/kg chlortetracycline; GOS, CON + 1500 mg/kg GOS.

**Figure 4:**
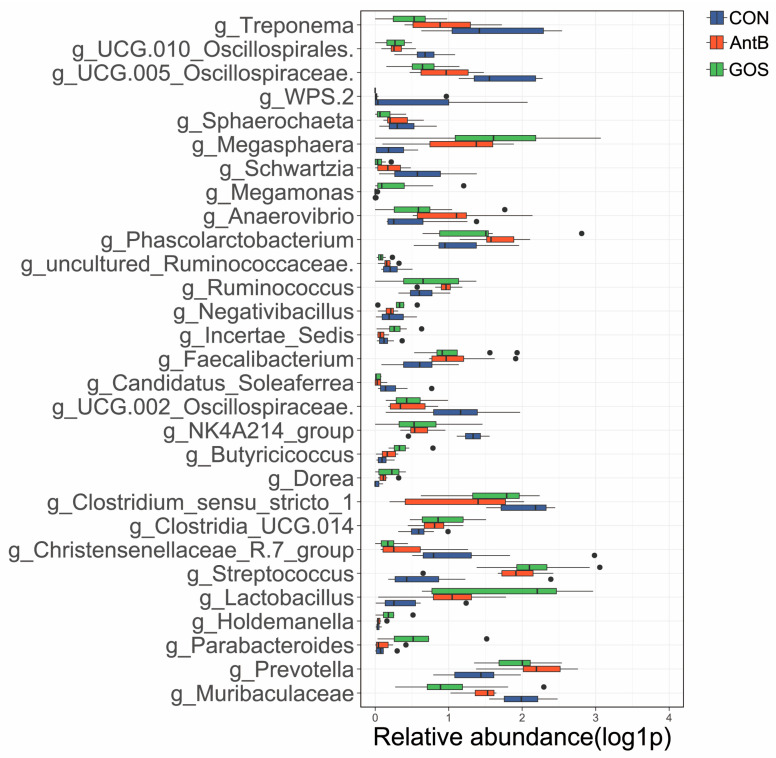
Identification of the most differentially abundant genera in the digesta samples of weaning piglets by linear discriminant analysis effect size (LEfSe). Genus-level difference bacteria. CON, corn–soybean meal-based diet; AntB, corn–soybean meal-type diet + 75 mg/kg chlortetracycline; GOS, CON + 1500 mg/kg GOS.

At the phylum level, it was observed that Firmicutes and Bacteroidota were the predominant phyla across all groups, with Firmicutes exhibiting the highest relative abundance. In contrast, Actinobacteria, Proteobacteria, and Spirochaetota constituted a relatively minor proportion of the microbial community. Notably, when comparing the GOS group to both the CON and AntB groups, an increase in the relative abundance of Firmicutes was recorded, while a decrease in the relative abundance of Bacteroidetes and Spirochaeta was noted (Figure 3A). At the genus level, an analysis of the ten most abundant bacterial genera revealed that the CON group primarily harbored microorganisms from Oscillospiraceae_UCG.005, Muribaculaceae, Clostridium_sensu_stricto_1, unclassified_Prevotellaceae, and unclassified_Lachnospiraceae. When comparing the CON group to the GOS and AntB groups, an increase in the relative abundance of Lactobacillus and Prevotella was identified, alongside a decrease in Clostridium_sensu_stricto_1 and Muribaculaceae. It is noteworthy that Lactobacillus was significantly more abundant in the GOS group than in the AntB group (Figure 3B). Furthermore, to elucidate specific value-added microbial species among the groups, a comparative analysis yielded a total of 29 different bacteria at the genus level. The CON group showed specific enrichment in genera such as g_Treponema, g_UCG.010_Oscillospirales, g_UCG.005_Oscillospiraceae, and others. Conversely, the AntB group exhibited significant proliferation of g_Anaerovibrio, g_Phascolarctobacterium, and g_Ruminococcus. The GOS group, on the other hand, demonstrated marked enrichment in g_Megasphaera, g_Megamonas, g_Faecalibacterium, and g_Lactobacillus, among others (Figure 4).

## 4. Discussion

In the present study, we thoroughly investigated the effects of dietary supplementation of galacto-oligosaccharides (GOS) on growth performance, intestinal morphology, and the overall intestinal health of weaned piglets. To achieve this, we employed high-throughput sequencing techniques to analyze the V3–V4 region of the 16S rRNA gene, thereby allowing for the precise monitoring of the colon microbiota in piglets subjected to either chlortetracycline (CTC) or GOS supplementation. Our findings suggest that dietary GOS supplementation exerts a favorable influence on the intestinal health of piglets by modulating the composition of gut microbiota. This modulation is characterized by an increase in the abundance of beneficial bacteria and a corresponding decrease in the proportion of potentially harmful bacteria. Furthermore, we hypothesize that the observed enhancement in growth performance may be intricately linked to the characteristics of the intestinal ecosystem. Previous research corroborates our findings, indicating that GOS serves as an effective additive for improving the growth performance of various animal species. For instance, Tian et al. (2018) demonstrated that piglets receiving diets supplemented with GOS exhibited a greater average daily gain (ADG) compared to those on a basal diet during the third week of the study [26]. Our results align with these findings, suggesting that the dietary inclusion of GOS has a beneficial impact on the growth performance of weaned piglets when compared to the control (CON) group.

The production performance of weaned piglets receiving CTC supplementation was observed to surpass that of the CON group; however, no statistically significant difference was noted between the antibiotic (AntB) group and the GOS group. Additionally, numerous studies have documented that the incorporation of prebiotic GOS into broiler diets enhances both growth rate and feed conversion efficiency, particularly when contrasted with calorie-matched control diets [32]. Moreover, GOS supplementation has been shown to incrementally increase final body weight, average daily gain, and feed efficiency in heifer calves [33]. The immature immune system resulting from the physiological dysfunction of the gastrointestinal tract in weaned piglets often precipitates diarrhea. Previous investigations have established that the addition of GOS to the diet significantly enhances growth performance while concurrently reducing the incidence of diarrhea in both broiler chickens and weaned piglets [32,34]. Chang et al. (year) further reported that GOS supplementation markedly diminished the incidence of diarrhea in calves [35]. The results of our study are consistent with the aforementioned literature, suggesting that the inclusion of GOS in the diets of weaned piglets effectively improves growth performance and mitigates the diarrhea rate.

The intestinal morphological index serves as a critical standard for evaluating the digestive and absorptive capacity of intestinal nutrients. Prebiotics, such as GOS, have been reported to promote growth by enhancing nutrient absorption through improvements in intestinal structure [36]. In our study, we observed that GOS supplementation led to a significant increase in the villus height of the jejunum and a marked enhancement in the ratio of villus height to crypt depth when compared to the CON group. Notably, the results indicated that the supplementation of two-day-old piglets with GOS over a three-day period significantly increased both villi height and surface area in the duodenum, while a 26-day supplementation significantly enhanced jejunal and duodenal villi height [16]. Furthermore, Lee et al. (year) reported that piglets receiving GOS exhibited significantly greater jejunal and ileal villi heights in comparison to their non-GOS-fed counterparts [27]. Given that villi are essential structures of the small intestine primarily responsible for nutrient absorption, an increase in villus height directly correlates with an enhanced surface area for nutrient uptake [37]. In conclusion, our study elucidates that GOS supplementation effectively sustains the morphological integrity of the small intestine in piglets, thereby enhancing their capacity for nutrient digestion and absorption while promoting overall production performance. The observed improvement in intestinal morphology may be attributed to the stimulatory effect of GOS supplementation on the proliferation of *Lactobacillus* spp. Previous studies have demonstrated that *Lactobacillus* can enhance villus height and increase the villus height-to-crypt depth ratio in the small intestine of piglets [38,39]. Additionally, we observed an increase in the number of goblet cells within the colonic crypts and mucosal layer thickness in the GOS treatment group, surpassing that of the CON group. Goblet cells are integral to the production of mucin glycoprotein, a vital component of the intestinal mucus barrier, serving as the primary natural defense against pathogens and harmful substances [40,41]. The increase in goblet cell numbers consequently signifies that GOS supplementation bolsters the chemical barrier within the colon. This morphological enhancement of the large intestine is likely attributable to improved butyrate production, a short-chain fatty acid primarily derived from microbial fermentation of dietary fibers, which fulfills approximately 70% of the energy requirements for colonocytes [42,43]. Furthermore, the literature suggests that butyrate may alleviate intestinal mucosal damage induced by pathogenic bacteria, thereby enhancing intestinal structural integrity and function [44]. Our findings indicate that the observed enrichment of genera such as g_Faecalibacterium and g_Butyricicoccus in the GOS group may be instrumental in elevating butyrate levels. Thus, we posit that GOS supplementation represents a promising strategy for enhancing intestinal morphology and protecting the intestinal barrier function in swine.

Gut microbes are integral to numerous physiological processes, and the composition of gut flora is pivotal for gut health and the development of the immune system [45,46]. The present study employs high throughput 16S-rDNA sequencing to evaluate the abundance and diversity of the colon microbial composition in piglets, thereby elucidating the impact of dietary interventions on gut microbiota. This study did not find statistical differences in the alpha diversity indices, specifically the Shannon and Richness indices, of the colon microbiota between the treatment groups. However, principal coordinate analysis (PCoA), utilized for beta diversity analysis, revealed distinct clustering of microbial communities between the control (CON) and the galacto-oligosaccharides (GOS) treatment groups. This finding suggests that GOS has a regulatory effect on the gut microbiota composition.

At the phylum level, Firmicutes, Bacteroidetes, Proteobacteria, and Actinobacteria are considered core constituents of the porcine gastrointestinal tract microbiome [47]. Our analysis indicates that the relative abundance of Firmicutes was significantly higher, while that of Bacteroidetes was notably lower in piglets receiving GOS. This alteration resulted in an enhanced Firmicutes to Bacteroidetes ratio, which is recognized as beneficial for gut health due to the SCFA-producing capabilities of Firmicutes [48]. The role of Bacteroidetes, primarily in carbohydrate degradation, appears to have a negative correlation with the daily weight gain of piglets [49,50]. Specifically, in weaned piglets, a higher Firmicutes to Bacteroidetes ratio has been observed in heavier individuals compared to their lighter counterparts [51]. This correlation suggests that an increased ratio may facilitate the host’s capacity for energy absorption and storage [52], a finding that aligns with previous studies indicating that a higher Firmicutes to Bacteroidetes ratio is associated with obesity and enhanced caloric absorption [53].

The introduction of GOS into the diet was also observed to significantly alter the relative abundances of specific bacterial genera [54]. Notably, GOS supplementation resulted in a marked increase in the populations of Faecalibacterium and Lactobacillus, alongside a reduction in Muribaculaceae. Faecalibacterium plays a crucial role in butyrate synthesis, which serves as a primary nutrient for the regeneration and repair of gut epithelial cells [55]. Lactobacillus, a dominant genus within the Firmicutes phylum, is known for its numerous health benefits, including pathogen exclusion, immunomodulation, and the production of beneficial metabolites [56]. Furthermore, Lactobacilli are capable of metabolizing carbohydrates, such as oligosaccharides and starch, which are subsequently fermented in the large intestine to produce SCFAs, thereby being utilized by the host [57]. Current research suggests that the inclusion of GOS in feed augments the abundance of Lactobacilli in weaned piglets while concurrently reducing the population of Escherichia coli, thereby enhancing growth performance [27,58]. We posit that the observed increase in Lactobacillus abundance may facilitate improvements in intestinal morphology, thereby promoting overall piglet growth. This hypothesis is corroborated by findings from Li et al., who documented that Lactobacillus reuteri significantly improved villus height and the villus to crypt (V/C) ratio in the jejunum of weaned piglets [59]. In addition to the aforementioned genera, the study revealed that GOS supplementation led to an increase in the relative abundance of Megasphaera and Clostridia_UCG_014, while it decreased the populations of Clostridium_sensu_stricto_1 and Treponema. Megasphaera contributes to large intestine functionality and effectively mitigates hyper-lactate accumulation-related diarrhea [60,61,62]. Conversely, certain Clostridium species are involved in butyrate synthesis, which is beneficial for intestinal growth and nutrient absorption [63,64,65]. Notably, Clostridium_sensu_stricto_1 has been linked to epithelial inflammation within the intestinal mucosa [66,67], while Treponema is associated with various diseases affecting the skin and mucosal membranes in multiple mammals [67,68]. In summary, the findings of this study indicate that the incorporation of GOS into the diets of weaned piglets effectively reduces the abundance of pathogenic bacterial strains while significantly increasing the populations of beneficial bacteria. Compared to the control group, the effects of GOS were comparable to those of traditional antibiotics; however, the beneficial bacteria populations in the GOS group surpassed those in the antibiotic group, thereby diminishing the risk of antibiotic resistance. Thus, the results strongly support the notion that GOS supplementation may enhance the disease resistance of piglets and provide a protective effect on intestinal health, serving as a viable alternative to in-feed antibiotics for maintaining a favorable gut microbiota composition. Nevertheless, further research is warranted to elucidate the underlying mechanisms by which GOS influences host intestinal microbiota.

## 5. Conclusions

In conclusion, this study posits that dietary galacto-oligosaccharides (GOS) can significantly enhance growth performance and intestinal morphology, while also modulating the relative abundance of specific bacterial populations by altering the overarching microbial structure. Notably, the augmented presence of Lactobacillus alongside the diminished prevalence of Clostridium_sensu_stricto_1 and Treponema in GOS-fed piglets may constitute a beneficial growth-promoting characteristic. Consequently, GOS presents a promising alternative to traditional in-feed antibiotics for weaned piglets within contemporary husbandry practices.

## Figures and Tables

**Table 1:** Composition and nutrient levels of the experimental diet (%, as-fed basis).

Items	Content (%)
Ingredients	
Corn	52.53
Soybean meal	19.00
Full-fat soybean powder	9.00
Fish meal	4.00
Whey powder	8.00
Soybean oil	3.60
Dicalcium phosphate	0.90
L-Lysine-HCl, 78%	0.46
L-Threonine	0.15
DL-Methionine	0.10
L-Tryptophan	0.03
L-Valine	0.12
Salt	0.30
Limestone	0.81
Premix ^a^	1.00
Total	100.00
Calculated nutrients	
Digestible energy (MJ/kg)	14.53
Crude protein	19.38
SID Lys	1.35
SID Met	0.39
SID Thr	0.72
SID Try	0.22

^a^ The premix provided the following (per kilogram of complex feed): Vitamin A, 12,000 IU; Vitamin D, 2500 IU; Vitamin E, 30 IU; Vitamin B12, 12 µg; Vitamin K, 3 mg; d-pantothenic acid, 15 mg; nicotinic acid, 40 mg; choline chloride, 400 mg; Mn, 40 mg; Zn, 100 mg; Fe, 90 mg; Cu, 8.8 mg; I, 0.35 mg; Se, 0.3 mg.

**Table 2:** Effects of GOS on growth performance and diarrhea rate of weaning piglets.

Items	CON	AntB	GOS	SEM	*p* Value
d 0 BW, kg	7.75	7.76	7.79	0.06	0.89
d 7 BW, kg	9.00 ^b^	9.26 ^a^	9.27 ^a^	0.06	0.01
d 14 BW, kg	10.92 ^b^	11.50 ^a^	11.53 ^a^	0.07	<0.01
Diarrhea rate, %	19.64 ^a^	8.04 ^b^	9.82 ^b^	3.20	0.047
d 1~7					
ADG, g/d	179 ^b^	214 ^a^	212 ^a^	2.33	<0.01
ADFI, g/d	336	358	359	17.74	0.59
G:F	0.55	0.61	0.60	0.04	0.48
d 7~14					
ADG, g/d	274 ^b^	319 ^a^	323 ^a^	5.94	<0.01
ADFI, g/d	639	615	619	15.18	0.53
G:F	0.43 ^b^	0.52 ^a^	0.52 ^a^	0.01	<0.01
d 1~14					
ADG, g/d	227 ^b^	267 ^a^	268 ^a^	2.56	<0.01
ADFI, g/d	487	486	489	9.27	0.98
G:F	0.47 ^b^	0.55 ^a^	0.55 ^a^	0.01	<0.01

Values are means, n = 8. SEM: standard error of the mean. Different superscripts within a row mean significantly different (*p* < 0.05). Abbreviations: ADFI, average daily feed intake; ADG, average daily weight gain; G:F, gain:feed; F/G, feed conversion ratio, the ratio of ADFI to ADG;CON, the basal diet group; AntB, the Antibiotic group; GOS, the galactose oligosaccharide group.

## Data Availability

The original contributions presented in this study are included in the article. Further inquiries can be directed to the corresponding author(s).

## References

[1] Gresse R., Chaucheyras-Durand F., Fleury M.A., Van de Wiele T., Forano E., Blanquet-Diot S. (2017). Gut Microbiota Dysbiosis in Postweaning Piglets: Understanding the Keys to Health. Trends Microbiol..

[2] Wang T., Teng K., Liu Y., Shi W., Zhang J., Dong E., Zhang X., Tao Y., Zhong J. (2019). *Lactobacillus plantarum* PFM 105 Promotes Intestinal Development Through Modulation of Gut Microbiota in Weaning Piglets. Front. Microbiol..

[3] Cromwell G.L. (2002). Why and how antibiotics are used in swine production. Anim. Biotechnol..

[4] Wijtten P.J., van der Meulen J., Verstegen M.W. (2011). Intestinal barrier function and absorption in pigs after weaning: A review. Br. J. Nutr..

[5] Hu Q., Liu C., Zhang D., Wang R., Gao F. (2020). Effects of Low-Dose Antibiotics on Gut Immunity and Antibiotic Resistomes in Weaned Piglets. Front. Immunol..

[6] Yang H., Paruch L., Chen X.J., Eerde A.V., Skomeda H., Wang Y.L., Liu D., Clarke J.L. (2019). Antibiotic Application and Resistance in Swine Production in China: Current Situation and Future Perspectives. Front. Vet. Sci..

[7] Neuman H., Forsythe P., Uzan A., Avni O., Koren O. (2018). Antibiotics in early life: Dysbiosis and the damage done. FEMS Microbiol. Rev..

[8] Toutain P.L., Ferran A.A., Bousquet-Melou A., Pelligand L., Lees P. (2016). Veterinary Medicine Needs New Green Antimicrobial Drugs. Front. Microbiol..

[9] Herrero-Fresno A., Zachariasen C., Hansen M.H., Nielsen A., Hendriksen R.S., Nielsen S.S., Olsen J.E. (2016). Apramycin treatment affects selection and spread ofa multidrug-resistant *Escherichia coli* strain able to colonize the human gut in the intestinal microbiota of pigs. Vet. Res..

[10] Smith M.G., Jordan D., Chapman T.A., Chin J.C., Barton M.D., Do T.N., Fahy V., Fairbrother J., Trott D. (2010). Antimicrobial resistance and virulence gene profiles in multi-drug resistant enterotoxigenic *Escherichia coli* isolated from pigs with post-weaning diarrhoea. Vet. Microbiol..

[11] Wang Y., Xu C., Zhang R., Chen Y., Shen Y., Hu F., Liu D., Lu J., Guo Y., Xia X. (2020). Changes in colistin resistance and mcr-1 abundance in *Escherichia coli* of animal and human origins following the ban of colistin-positive additives in China: An epidemiological comparative study. Lancet Infect. Dis..

[12] Gibson G.R., Hutkins R., Sanders M.E., Prescott S.L., Reimer R.A., Salminen S.J., Reid G. (2017). Expert consensus document: The International Scientific Association for Probiotics and Prebiotics (ISAPP) consensus statement on the definition and scope of prebiotics. Nat. Rev. Gastroenterol. Hepatol..

[13] Callaway T.R., Edrington T.S., Anderson R.C., Harvey R.B., Genovese K.J., Kennedy C.N., Venn D.W., Nisbet D.J. (2008). Probiotics, prebiotics and competitive exclusion for prophylaxis against bacterial disease. Anim. Health Res. Rev..

[14] Mikkelsen L.L., Jakobsen M., Jensen B.B. (2003). Effects of dietary oligosaccharides on microbial diversity and fructo-oligosaccharide degrading bacteria in faeces of piglets post-weaning. Anim. Feed Sci. Technol..

[15] Mair C., Plitzner C., Domig K.J., Schedle K., Windisch W. (2010). Impact of inulin and a multispecies probiotic formulation on performance, microbial ecology and concomitant fermentation patterns in newly weaned piglets. J. Anim. Physiol. Anim. Nutr..

[16] Alizadeh A., Akbari P., Difilippo E., Schols H.A., Ulfman L.H., Schoterman M.H.C., Braber S. (2016). The piglet as a model for studying dietary components in infant diets: Effects of galacto-oligosaccharides on intestinal functions. Br. J. Nutr..

[17] Samanta A.K., Jayapal N., Jayaram C., Roy S., Kolte A.P., Senani S., Sridhar M. (2015). Xylooligosaccharides as prebiotics from agricultural by-products: Production and applications. Bioact. Carbohydr. Diet. Fibre.

[18] Hill A., Tian F., Karboune S. (2016). Synthesis of Levan and Fructooligosaccharides by Levansucrase: Catalytic, Structural and Substrate-Specificity Properties. Curr. Org. Chem..

[19] Tzortzis G. (2009). Functional properties of the second generation prebiotic Galacto-oligosaccharide (B-GOS). Agro Food Ind. Hi Tech..

[20] Vandenplas Y., Zakharova I., Dmitrieva Y. (2015). Oligosaccharides in infant formula: More evidence to validate the role of prebiotics. Br. J. Nutr..

[21] Boehm G., Jelinek J., Stahl B., Van Laere K., Knol J., Fanaro S., Vigi V. (2004). Prebiotics in infant formulas. J. Clin. Gastroenterol..

[22] Martinez-Ferez A., Rudloff S., Guadix A., Henkel C.A., Pohlentz G., Boza J.J., Guadix E.M., Kunz C. (2006). Goats’ milk as a natural source of lactose-derived oligosaccharides: Isolation by membrane technology. Int. Dairy J..

[23] Cardellecobas A., Corzo N., Olano A., Peláez C., Requena T., Ávila M. (2011). Galactooligosaccharides derived from lactose and lactulose: Influence of structure on lactobacillus, streptococcus and bifidobacterium growth. Int. J. Food Microbiol..

[24] Gormley A., Garavito-Duarte Y., Kim S.W. (2024). The Role of Milk Oligosaccharides in Enhancing Intestinal Microbiota, Intestinal Integrity, and Immune Function in Pigs: A Comparative Review. Biology.

[25] Farthing M.J. (2004). Bugs and the gut: An unstable marriage. Best Pract. Res. Clin. Gastroenterol..

[26] Tian S., Wang J., Yu H., Wang J., Zhu W. (2018). Effects of galacto-oligosaccharides on growth and gut function of newborn suckling piglets. J. Anim. Sci. Biotechnol..

[27] Lee A., Mansbridge S.C., Liang L., Connerton I.F., Mellits K.H. (2023). Galacto-Oligosaccharides Increase the Abundance of Beneficial Probiotic Bacteria and Improve Gut Architecture and Goblet Cell Expression in Poorly Performing Piglets, but Not Performance. Animals.

[28] Boston T.E., Wang F., Lin X., Kim S.W., Fellner V., Scott M.F., Odle J. (2024). Prebiotic galactooligosaccharide improves piglet growth performance and intestinal health associated with alterations of the hindgut microbiota during the peri-weaning period. J. Anim. Sci. Biotechnol..

[29] NRC (2012). Nutrient Requirements of Swine.

[30] Song Y., Luo Y., Yu B., He J., Zheng P., Mao X., Yu J. (2021). Tannic acid extracted from gallnut prevents post-weaning diarrhea and improves intestinal health of weaned piglets. Anim. Nutr..

[31] Shatos M.A., Ríos J.D., Horikawa Y., Hodges R.R., Chang E.L., Bernardino C.R., Dartt D.A. (2003). Isolation and Characterization of Cultured Human Conjunctival Goblet Cells. Investig. Ophthalmol. Vis. Sci..

[32] Richards P.J., Flaujac Lafontaine G.M., Connerton P.L., Liang L., Asiani K., Fish N.M., Connerton I.F. (2020). Galacto-oligosaccharides modulate the juvenile gut microbiome and innate immunity to improve broiler chicken performance. Msystems.

[33] Yu X., Ma F., Dai H., Liu J., Hashem N.M., Sun P. (2023). Effects of Different Galacto-Oligosaccharide Supplementation on Growth Performance, Immune Function, Serum Nutrients, and Appetite-Related Hormones in Holstein Calves. Animals.

[34] Tian S., Wang J., Wang J., Zhu W. (2022). Differential Effects of Early-Life and Postweaning Galacto-oligosaccharide Intervention on Colonic Bacterial Composition and Function in Weaning Piglets. Appl. Environ. Microbiol..

[35] Chang M., Wang F., Ma F., Jin Y., Sun P. (2022). Supplementation with galacto-oligosaccharides in early life persistently facilitates the microbial colonization of the rumen and promotes growth of preweaning Holstein dairy calves. Anim. Nutr..

[36] Pan L., Mohammed F., Qin G., Yuan Z., Bao N. (2018). The influences of soybean agglutinin and functional oligosaccharides on the intestinal tract of monogastric animals. Int. J. Mol. Sci..

[37] Choe D.W., Loh T.C., Foo H.L., Hair-Bejo M., Awis Q.S. (2012). Egg production, faecal pH and microbial population, small intestine morphology, and plasma and yolk cholesterol in laying hens given liquid metabolites produced by *Lactobacillus plantarumstrains*. Br. Poult. Sci..

[38] Wang Q., Sun Q., Qi R., Wang J., Qiu X., Liu Z., Huang J. (2019). Effects of *Lactobacillus plantarum* on the intestinal morphology, intestinal barrier function and microbiota composition of suckling piglets. J. Anim. Physiol. Anim. Nutr..

[39] Zhu C., Yao J., Zhu M., Zhu C., Yuan L., Li Z., Liu H.Y. (2022). A meta-analysis of Lactobacillus-based probiotics for growth performance and intestinal morphology in piglets. Front. Vet. Sci..

[40] Wang W., Chen Y., Wang J., Lv Z., Li E., Zhao J., Liu L., Wang F., Liu H. (2022). Effects of reduced dietary protein at high temperature in summer on growth performance and carcass quality of finishing pigs. Animals.

[41] Gustafsson J.K., Johansson M.E.V. (2022). The role of goblet cells and mucus in intestinal homeostasis. Nat. Rev. Gastroenterol. Hepatol..

[42] Jacobi S.K., Odle J. (2012). Nutritional factors influencing intestinal health of the neonate. Adv. Nutr..

[43] Koh A., De Vadder F., Kovatcheva-Datchary P., Bäckhed F. (2016). From dietary fiber to host physiology: Short-chain fatty acids as key bacterial metabolites. Cell.

[44] Chen J., Xu Q., Li Y., Tang Z., Sun W., Zhang X., Sun Z. (2019). Comparative effects of dietary supplementations with sodium butyrate, medium-chain fatty acids, and n-3 polyunsaturated fatty acids in late pregnancy and lactation on the reproductive performance of sows and growth performance of suckling piglets. J. Anim. Sci..

[45] Hu J., Chen L., Zheng W., Shi M., Liu L., Xie C., Yan X. (2018). *Lactobacillus frumenti* Facilitates Intestinal Epithelial Barrier Function Maintenance in Early Weaning Piglets. Front. Microbiol..

[46] Nowland T.L., Plush K.J., Barton M., Kirkwood R.N. (2019). Development and Function of the Intestinal Microbiome and Potential Implications for Pig Production. Animals.

[47] Wang X., Tsai T., Deng F., Wei X., Chai J., Knapp J. (2019). Longitudinal investigation of the swine gut microbiome from birth to market reveals stage and growth performance associated bacteria. Microbiome.

[48] Gao R., Tian S., Wang J., Zhu W. (2021). Galacto-oligosaccharides improve barrier function and relieve colonic inflammation via modulating mucosa-associated microbiota composition in lipopolysaccharides-challenged piglets. J. Anim. Sci. Biotechnol..

[49] Ban-Tokuda T., Maekawa S., Miwa T., Ohkawara S., Matsui H. (2017). Changes in faecal bacteria during fattening in finishing swine. Anaerobe.

[50] Spence C., Wells W.G., Smith C.J. (2006). Characterization of the primary starch utilization operon in the obligate anaerobe *Bacteroides fragilis*: Regulation by carbon source and oxygen. J. Bacteriol..

[51] Han G.G., Lee J.Y., Jin G.D., Park J., Choi Y.H., Chae B.J., Choi Y.J. (2017). Evaluating the association between body weight and the intestinal microbiota of weaned piglets via 16S rRNA sequencing. Appl. Microbiol. Biotechnol..

[52] Magnusson K.R., Hauck L., Jeffrey B.M., Elias V., Humphrey A., Nath R., Bermudez L.E. (2015). Relationships between diet-related changes in the gut microbiome and cognitive flexibility. Neuroscience.

[53] Guida S., Venema K. (2015). Gut microbiota and obesity: Involvement of the adipose tissue. J. Funct. Foods.

[54] Ley R., Turnbaugh P., Klein S., Gordon J.I. (2006). Human Gut Microbes Associated with Obesity. Nature.

[55] Lopezsiles M., Duncan S.H., Garciagil L.J., Martinezmedina M. (2017). *Faecalibacterium prausnitzii*: From microbiology to diagnostics and prognostics. ISME J..

[56] Kravtsov E.G., Yermolayev A.V., Anokhina I.V., Yashina N.V., Chesnokova V.L., Dalin M.V. (2008). Adhesion characteristics of lactobacillus is a criterion of the probiotic choice. Bull. Exp. Biol. Med..

[57] Gänzle M.G., Follador R. (2012). Metabolism of oligosaccharides and starch in lactobacilli: A review. Front. Microbiol..

[58] Xing Y.-Y., Li K.-N., Xu Y.-Q., Wu Y.-Z., Shi L.-L., Guo S.-W., Yan S.-M., Jin X., Shi B.-L. (2020). Effects of galacto-oligosaccharide on growth performance, feacal microbiota, immune response and antioxidant capability in weaned piglets. J. Appl. Anim. Res..

[59] Li Y., Hou S., Chen J., Peng W., Wen W., Chen F., Huang X. (2019). Oral administration of *Lactobacillus delbrueckii* during the suckling period improves intestinal integrity after weaning in piglets. J. Funct. Foods.

[60] Dumonceaux T.J., Hill J.E., Hemmingsen S.M., Van Kessel A.G. (2006). Characterization ofintestinal microbiota and response to dietary virginiamycin supplementation in the broiler chicken. Appl. Environ. Microbiol..

[61] Lin J. (2011). Effect ofantibiotic growth promoters on intestinal microbiota in food animals: A novel model for studying the relationship between gut microbiota and human obesity?. Front. Microbiol..

[62] Ushida K., Kishimoto A., Piao S.J., Itoh M., Shiga A., Nakanishi N., Tsukahara T. (2009). An epidemiological survey on pigs showing symptoms of infectious enteric diseases and dyspepsia in Japan. Anim. Sci. J..

[63] Ariyoshi T., Hagihara M., Takahashi M., Mikamo H. (2022). Effect of *Clostridium butyricum* on gastrointestinal infections. Biomedicines.

[64] Wang Y., Xu L., Liu J., Zhu W., Mao S. (2017). A high grain diet dynamically shifted the composition ofmucosa-associated microbiota and induced mucosal injuries in the colon of sheep. Front. Microbiol..

[65] Ho E.L., Lukehart S.A. (2011). Syphilis: Using modern approaches to understand an old disease. J. Clin. Investig..

[66] Zhao J.B., Liu P., Huang C.F., Liu L., Li E.K., Zhang G., Zhang S. (2018). Effect of wheat bran on apparent total tract digestibility, growth performance, fecal microbiota and their metabolites in growing pigs. Anim. Feed. Sci. Technol..

[67] Oladele P., Li E., Lu H., Cozannet P., Nakatsu C., Johnson T., Adeola O., Ajuwon K.M. (2021). Effect of a carbohydrase admixture in growing pigs fed wheat-based diets in thermoneutral and heat stress conditions. J. Anim. Sci..

[68] Ma L., Wang H., Qiu Y., Bai Z., Yang Z., Li E., Ma X., Xiao D. (2024). Alternative Uses of Fermented Wheat Bran: A Mini Review. Fermentation.

